# Spin‐Orchestrated Lithium Diffusion in Reforged Ferromagnetic Fe@C Anodes

**DOI:** 10.1002/advs.202506133

**Published:** 2025-07-02

**Authors:** Myeong Seok Goh, Hyunsub Shin, Jaehun Lee, No‐Kuk Park, Joonwoo Kim, Sang Woo Joo, Ki Hyeon Kim, Misook Kang

**Affiliations:** ^1^ Department of Chemistry College of Natural Sciences Yeungnam University Gyeongsan Gyeongbuk 38541 Republic of Korea; ^2^ Institute of Clean Technology Yeungnam University Gyeongsan Gyeongbuk 38541 Republic of Korea; ^3^ Industrial Gas Research Group Research Institute of Industrial Science & Technology (RIST) 67 Cheongam‐ro, Nam‐gu Pohang Gyeongbuk 37673 Republic of Korea; ^4^ School of Mechanical Engineering Yeungnam University Gyeongsan Gyeongbuk 38541 Republic of Korea; ^5^ Department of Physics College of Natural Sciences Yeungnam University Gyeongsan 38541 Republic of Korea

**Keywords:** conductor‐free lithium‐ion battery, ferromagnetic Fe@C Anode, hydrogen pyrolysis‐derived electrode materials, in situ carbon encapsulation, spin‐guided lithium diffusion

## Abstract

This reports a dual‐functional approach in which Fe catalysts, initially employed for methane pyrolysis to generate COx‐free hydrogen, are directly repurposed as anode materials following in situ carbon deposition. During methane splitting, catalytic decomposition of CH₄ at 900 °C forms onion‐like graphitic carbon shells (≈280 layers) around Fe cores (≈50 nm), producing a structurally stable and electrically conductive Fe@C900 composite without post‐treatment. This carbon‐enriched catalyst demonstrates exceptional electrochemical behavior when transitioned into a battery context. Without any conductive additives, Fe@C900 delivers a reversible capacity of 380 mAh g⁻¹ with 98% retention over 1000 cycles at 1 C. Under a 5000 G magnetic field, spin alignment within the Fe cores triggers directional lithium‐ion migration, enhancing rate performance by 150%. Multimodal characterization reveals accelerated lithium kinetics, stable SEI evolution, and deep lithiation behavior. DFT calculations further confirm strong lithium adsorption (−24.14 eV) and low insertion barriers (−22.85 eV), validating the spin‐guided diffusion mechanism. This work introduces a new class of hydrogen‐derived ferromagnetic anodes, where the byproduct of a clean hydrogen process is re‐engineered into a high‐rate, conductor‐free lithium storage platform. By coupling hydrogen generation with energy storage through shared material intermediates, this strategy offers a scalable path to carbon‐efficient, magnetically enhanced battery systems.

## Graphical abstract

Upcycled Fe@C900 from methane pyrolysis delivers superior lithium storage performance. Conductor‐free Fe@C900 achieves 367 mAh g^−^¹ and 98% capacity retention over 1000 cycles at 1C. Expanded interlayer spacing and stable SEI architecture ensure long‐term electrochemical durability. The external magnetic field aligns Fe core spins, accelerating Li⁺ diffusion and charge‐transfer kinetics. DFT calculations reveal low insertion barriers and strong Li⁺ binding in spin‐active carbon shells.

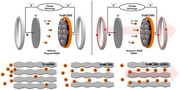

## 1 Introduction

The relentless expansion of electric vehicles (EVs), grid‐scale renewable energy storage, and portable electronics has sharply intensified the need for next‐generation lithium‐ion batteries (LIBs) with high‐rate capability, long‐term durability, and sustainable material sourcing. Among all battery components, the anode plays a pivotal role by dictating lithium‐ion diffusivity, charge‐storage capacity, Coulombic efficiency, and structural stability under rapid cycling conditions. Despite its ubiquity, commercial graphite suffers from inherent limitations, including a moderate theoretical capacity of 372 mAh g⁻¹, slow lithium intercalation kinetics, and pronounced polarization under fast‐charging—often leading to lithium plating and safety risks.^[^ ^1^ ^]^ Additionally, the layered structure of graphite undergoes mechanical degradation and unstable SEI evolution during prolonged cycling, culminating in capacity fade and reduced lifespan.^[^ ^2^ ^]^ To compensate for poor intrinsic conductivity, graphite anodes rely heavily on conductive additives (e.g., Super P, carbon black), which dilute the active material content, increase cost, and add processing complexity.^[^ ^3^ ^]^

These limitations have galvanized efforts to explore next‐generation anode materials that combine higher capacity, faster ion transport, and structural robustness without depending on auxiliary conductive networks. Among the promising alternatives, transition metal‐based systems—particularly iron oxides—stand out for their high theoretical capacities (>900 mAh g⁻¹), multiple redox centers, elemental abundance, and environmental benignity.^[^ ^4^, ^5^ ^]^ Recently, ferromagnetic Fe‐based nanostructures have garnered renewed interest not only for their energy density, but also for their ability to support spin‐polarized charge transport under external magnetic fields.^[^ ^6^, ^7^ ^]^ This emerging concept of spin‐guided lithium‐ion diffusion proposes that aligned magnetic domains within Fe cores generate anisotropic electronic fields that steer lithium‐ion migration, enhance charge‐transfer kinetics, and mitigate SEI degradation at high rates.

However, the translation of Fe‐based systems into practical LIB anodes remains hampered by several critical issues: poor intrinsic electronic conductivity, large volume expansion during cycling, and the need for complex, multi‐step synthesis routes involving hazardous chemicals or high‐energy input.^[^ ^8^, ^9^ ^]^ Carbon‐based hybrids—such as carbon onions, hollow carbon nanospheres, and hard carbon matrices—have been proposed to alleviate these challenges by enhancing conductivity and buffering structural stress.^[^ ^10^ ^]^ Yet, most require post‐synthesis graphitization or chemical activation steps that are resource‐intensive and poorly aligned with sustainable processing. Moreover, few of these approaches offer opportunities for integration with other clean energy technologies.

To overcome these limitations, we propose a dual‐functional material strategy that bridges hydrogen production and battery applications through a shared catalytic architecture. Specifically, we introduce a ferromagnetic Fe@C900 anode directly derived from carbon‐enriched iron catalysts used in methane pyrolysis, a thermocatalytic route to generate COx‐free hydrogen. During pyrolysis at 900 °C, Fe₂O₃ particles are reduced and concurrently coated with ≈280 graphitic carbon layers through in situ CH₄ decomposition, producing Fe@C nanoparticles encapsulated in onion‐like carbon shells without any post‐treatment. Originally engineered for turquoise hydrogen production, this carbon‐enriched catalyst is repurposed as a high‐performance LIB anode that operates entirely without conductive additives.

Remarkably, under a 5000 G magnetic field, the ferromagnetic Fe cores within Fe@C900 exhibit spin alignment that triggers directional lithium‐ion diffusion, resulting in a 150% increase in reversible capacity at 1 C. Even without conductive additives, the electrode achieves 380 mAh g⁻¹ with 98% retention over 1000 cycles. Multimodal characterization—GITT, EIS, in situ XRD/Raman, EFM, and MFM—confirms enhanced ionic transport, deeper lithiation, and uniform SEI formation under magnetic modulation.^[^ ^10^ ^]^ First‐principles calculations further validate the spin‐channeling mechanism, revealing superior lithium affinity (−24.14 eV) and a low energy barrier (−22.85 eV) for lithium insertion in Fe@C900, surpassing that of graphite.

This study pioneers a conductor‐free, spin‐functional anode system forged from hydrogen production catalysts, establishing a new magnetic‐diffusion paradigm for lithium‐ion transport. It simultaneously demonstrates the feasibility of converting carbon‐enriched catalytic residues into high‐performance energy materials, advancing material circularity and cross‐sector sustainability in electrochemical energy storage.

Please refer to the Supplementary Section for detailed procedures, including catalyst preparation, methane pyrolysis setup, material characterization, electrochemical testing, and magnetic field‐assisted measurements.

## 2 Results and Discussion

### 2.1 Upcycled Performance: Benchmarking Pyrolyzed Fe@Cx for Conductor‐Free Lithium Storage

**Figure 1:**
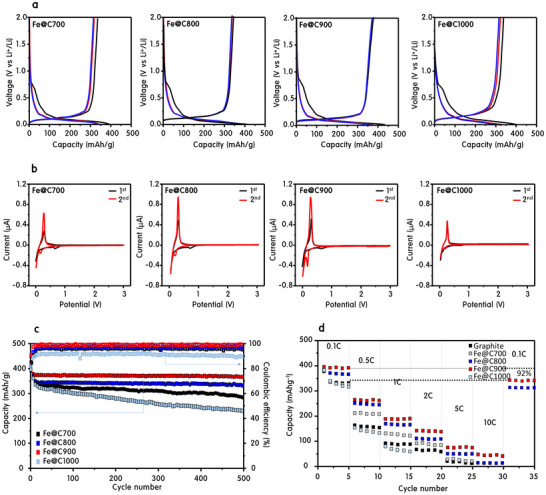
From waste to power: benchmarking upcycled Fe@C_x_ anodes for high‐efficiency lithium storage. a) Charge–discharge profiles of Fe@C_x_ anodes (Fe@C700, Fe@C800, Fe@C900, Fe@C1000) at 0.1 C, b) Cyclic voltammetry (CV) curves of Fe@C_x_ anodes at a scan rate of 0.1 mV s⁻¹, highlighting reversible Li⁺ insertion/extraction peaks, c) Long‐term cycling performance and Coulombic efficiency of Fe@C_x_ anodes over 500 cycles at 0.1 C, and d) Rate capability tests of Fe@C_x_ anodes at varying C‐rates (0.1 to 10 C).

**Figure** **1** and Figures S1, S2 (Supporting Information) comprehensively demonstrate the electrochemical superiority of Fe@Cx (x = 700–1000 °C) catalysts upcycled from methane‐splitting hydrogen systems. Among them, Fe@C900 exhibited the highest reversible capacity of 367 mAh g⁻¹ at 0.1 C (Figure 1a), outperforming other Fe@Cx variants—Fe@C700 (352), Fe@C800 (361), and Fe@C1000 (343)—and notably exceeding commercial graphite (340 mAh g⁻¹, with conductive additives; Figure S1a, Supporting Information). This performance is achieved without any conductive additives, nearly matching graphite's theoretical limit of 372 mAh g⁻¹.^[^ ^11^ ^]^ Cyclic voltammetry (Figure 1b) confirms superior redox reversibility in Fe@C900, which shows the most prominent and symmetric Li⁺ insertion/extraction peaks below 0.5 V. The progressive enhancement of redox peak intensity from Fe@C700 to Fe@C900 indicates improved Li⁺ kinetics and reduced polarization, aligning with prior reports of electrochemical enhancement through optimized hybrid carbon structures.^[^ ^12^ ^]^ Over 500 cycles at 0.1 C, Fe@C900 retained near‐complete capacity and Coulombic efficiency approaching 100%, while Fe@C700 and Fe@C1000 exhibited clear degradation (Figure 1c). High‐rate tests (Figure 1d) demonstrated Fe@C900's robustness under stress, retaining 50 mAh g⁻¹ at 10 C and recovering 92% of original capacity when cycled back to 0.1 C. Compared with graphite, Fe@C900 offered both higher capacity and better reversibility in CV curves (Figure S1b, Supporting Information), with stronger intercalation peaks and more stable kinetics despite lacking conductive additives. XRD analysis (Figure S2a, Supporting Information) revealed a left‐shifted (002) peak for Fe@C900, confirming expanded interlayer spacing, which facilitates deeper Li⁺ intercalation. Graphite, by contrast, follows a stepwise intercalation model (LiC₂₄ → LiC₁₂), stabilizing at Stage 2 under moderate conditions, as described by the Daumas–Herold model.^[^ ^13^ ^]^ Fe@C900, owing to its expanded spacing and open carbon channels, enables full lithiation to Stage 1 (LiC₆). Most critically, Fe@C900 maintained 98% capacity after 1000 cycles at 1 C (Figure S2b, Supporting Information), whereas commercial graphite degraded to ≈70%, and Fe@C1000 failed before 200 cycles. These results are consistent with prior observations of structural fatigue and SEI instability in conventional graphite anodes over extended cycling.^[^ ^14^ ^]^ In contrast, the highly ordered carbon shells in Fe@C900 suppress volume expansion and foster stable SEI formation, as also demonstrated in previous hybrid nanostructure systems.^[^ ^15^ ^]^ Collectively, these data confirm that Fe@C900 is a rare class of additive‐free, magnetically active, and structurally resilient anode materials, upcycled from hydrogen evolution catalysts and fully competitive with conventional graphite—both in performance and durability.

### 2.2 Crystallized Carbon Armor: Structural Refinement and Conductivity in Fe@C900

**Figure 2:**
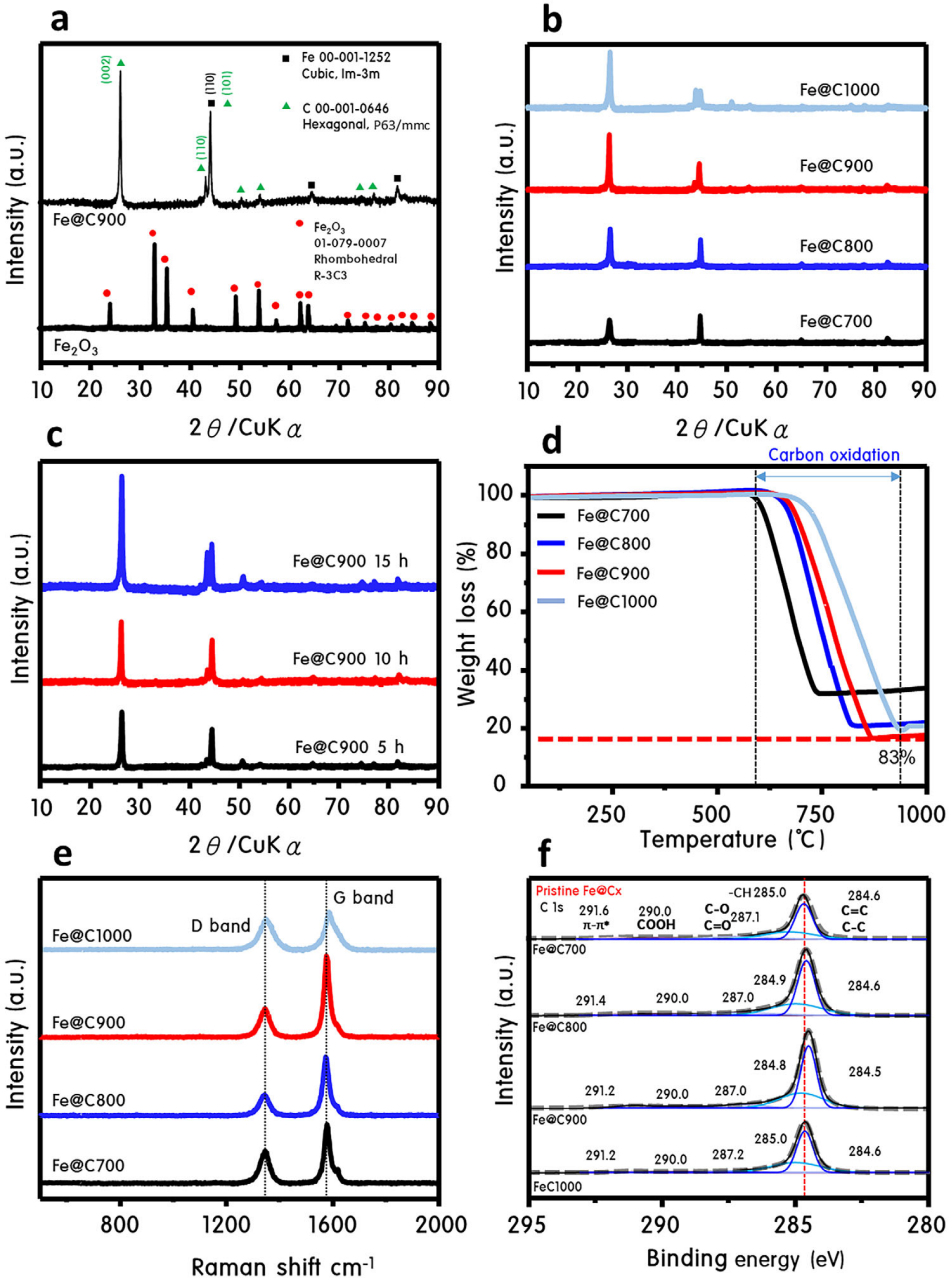
Forged in flame: structural fingerprint of methane‐pyrolyzed ferromagnetic carbon architectures. a) XRD patterns of Fe₂O₃ and Fe@C900, b) XRD patterns of Fe@C_x_ samples at various pyrolysis temperatures, c) XRD patterns of Fe@C900 at different pyrolysis durations, d) TGA analysis of Fe@C_x_ samples, e) Raman spectra of Fe@C_x_ samples, highlighting the D and G bands and f) XPS C 1s spectra of Fe@C_x_ samples.

**Figure 3:**
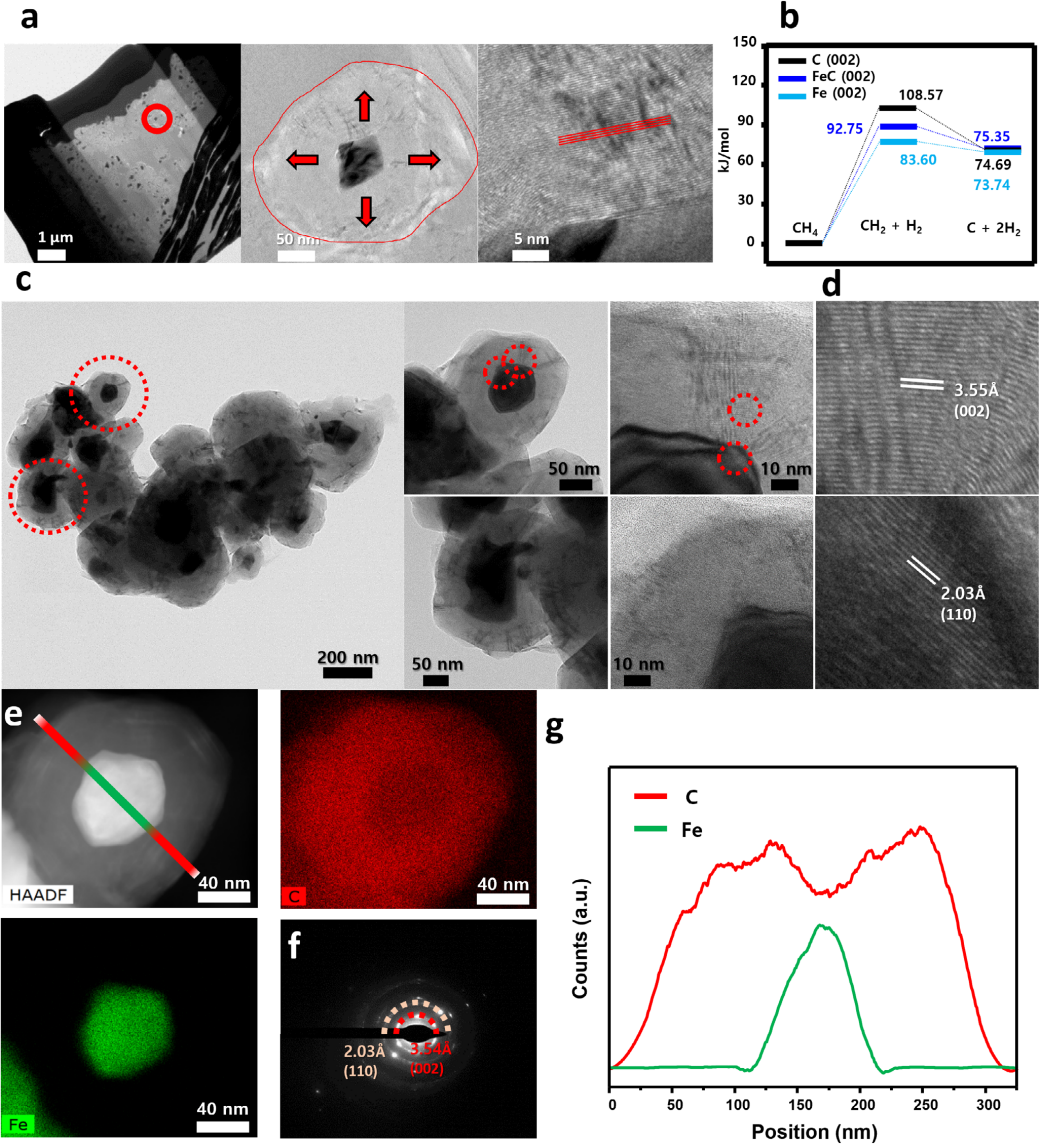
Inside the armor: core–shell geometry and interfacial definition of Fe@C900. a) FIB‐TEM and HR‐TEM images showing ≈50 nm Fe core and ≈100 nm carbon shell; red arrows indicate radial carbon growth. b) Gibbs free energy diagram for CH₄ decomposition on C, FeC, and Fe surfaces. c) TEM/HR‐TEM images showing intact multilayered carbon shells; red circles mark representative particles. d) Lattice fringes with d‐spacings of 3.55 Å (C (002)) and 2.03 Å (Fe (110)). e) HAADF‐STEM and EDS elemental maps of C and Fe; red–green line shows scan direction. f) SAED pattern indexed to graphitic carbon and metallic Fe planes. g) EDS line profile showing clear Fe‐core and C‐shell separation across the particle.

The structural and chemical evolution of Fe@Cx catalysts during methane pyrolysis is systematically illustrated in **Figure** **2**, establishing Fe@C900 as the optimal architecture for lithium‐ion storage. XRD analysis (Figure 2a–c) shows that pristine Fe₂O₃ exhibits a rhombohedral structure (*R‐3C3*), which transforms into cubic Fe (*Im‐3m*) and hexagonal graphitic carbon (*P63/mmc*) upon pyrolysis.^[^ ^16^, ^17^, ^18^ ^]^ As the pyrolysis temperature increases, the (002) carbon peak intensifies, reflecting enhanced graphitization along the Z‐axis. At 900 °C, the emergence and sharpening of the (100) peak signify well‐aligned lateral growth, indicating the formation of highly crystalline, hexagonal carbon layers. Thermal analysis by TGA (Figure 2d) reveals carbon oxidation initiating ≈620 °C and completing near 920 °C. Fe@C900 exhibits the highest carbon content (≈83 wt%), confirming that 900 °C is the optimal condition for uniform and thick carbon shell formation. Raman spectroscopy (Figure 2e) further supports this, with Fe@C900 showing the lowest ID/IG ratio among the series—indicative of minimal disorder and superior graphitization.^[^ ^19^, ^20^ ^]^ In contrast, Fe@C1000 displays a broadened D‐band and elevated ID/IG, pointing to thermal overstress and carbon structure degradation.^[^ ^21^ ^]^ XPS analysis (Figure 2f) highlights dominant sp^2^‐carbon peaks at 284.6 eV, with Fe@C900 exhibiting the largest peak area and clearest *π–π* ^*^ transitions (291.2 eV), confirming its advanced graphitic order.^[^ ^22^, ^23^ ^]^ The gradual disappearance of oxygen‐containing groups (C═O, COOH) with increasing pyrolysis temperature^[^ ^24^ ^]^ validates successful deoxygenation. No Fe 2p signal is detected, confirming that the Fe cores are fully encapsulated within carbon layers thicker than 100 nm, which act as a robust chemical shield. The core–shell architecture and structural formation mechanism of Fe@C900 were comprehensively validated through a multi‐modal characterization approach, including FIB‐TEM, HR‐TEM, HAADF‐STEM, and spatially resolved EDS line profiling (**Figure** **3**). As shown in Figure 3a, the focused ion beam (FIB)‐assisted TEM imaging reveals a well‐defined core–shell structure comprising a ≈50 nm metallic Fe core encapsulated within a ≈100 nm onion‐like carbon shell. The radial alignment of graphitic layers, marked by red arrows, strongly supports a catalytically driven shell growth mechanism originating at the Fe surface. This growth mechanism is thermodynamically rationalized in Figure 3b, where DFT‐calculated Gibbs free energy diagrams indicate that CH₄ decomposition is energetically most favorable on bare Fe surfaces. As FeC and carbon layers accumulate, the catalytic activity progressively declines, resulting in self‐limiting lateral thickening and eventual shell damage at high temperature (1000 °C, Figure S3, Supporting Information), consistent with observed mechanical degradation. Figure 3c provides both low‐ and high‐magnification TEM images confirming the concentric, multilayered carbon shell surrounding each Fe nanoparticle. In Figure 3d, high‐resolution lattice fringes reveal interlayer spacings of 3.55  and 2.03 Å, corresponding to the (002) plane of graphitic carbon and the (110) plane of bcc Fe, respectively—validating the high crystallinity of both core and shell. Critically, the elemental distinction between the Fe core and carbon shell is clearly established in Figure 3e via HAADF‐STEM imaging and EDS elemental mapping. Fe is strictly confined to the core region, while carbon occupies the entire shell domain. To further quantify the interface, a line‐scan elemental profile (Figure 3g, along the red–green axis in 3e) was performed. The results show a sharp peak in Fe intensity at the core center that rapidly declines at the shell boundary, while the C signal remains uniformly strong across the shell, conclusively verifying a spatially separated core–shell arrangement. Finally, the SAED pattern in Figure 3f exhibits distinct diffraction rings indexed to the (002) plane of graphitic carbon and the (110) plane of Fe, corroborating the presence of well‐defined crystalline domains. Taken together, these multi‐technique results unequivocally confirm the formation of a robust, compositionally distinct Fe@C900 core–shell nanostructure. This architecture plays a crucial role in enhancing the mechanical integrity and preserving interfacial stability during electrochemical cycling, particularly under external magnetic fields. BET surface area analysis (Figure S4, Supporting Information) shows that commercial graphite has a low surface area (8.415 m^2^ g^−1^) and no observable mesoporosity. In contrast, Fe@C900 exhibits a pronounced Type IV isotherm with a large hysteresis loop and surface area of 22.676 m^2^ g^−1^, indicating a well‐connected mesoporous framework.^[^ ^25^ ^]^ Fe@C1000 shows a collapsed structure and surface area loss, again emphasizing Fe@C900's balance of porosity and thermal stability. Collectively, these data establish Fe@C900 as the structurally optimal form among all Fe@Cx catalysts—offering a crystalline, defect‐minimized, and highly conductive carbon armor that supports fast lithium transport, stable solid electrolyte interphase (SEI) formation, and long‐term cycling performance in electrochemical systems.

### 2.3 Surface Matters: How Spin and Shell Rewire SEI Stability

**Figure 4:**
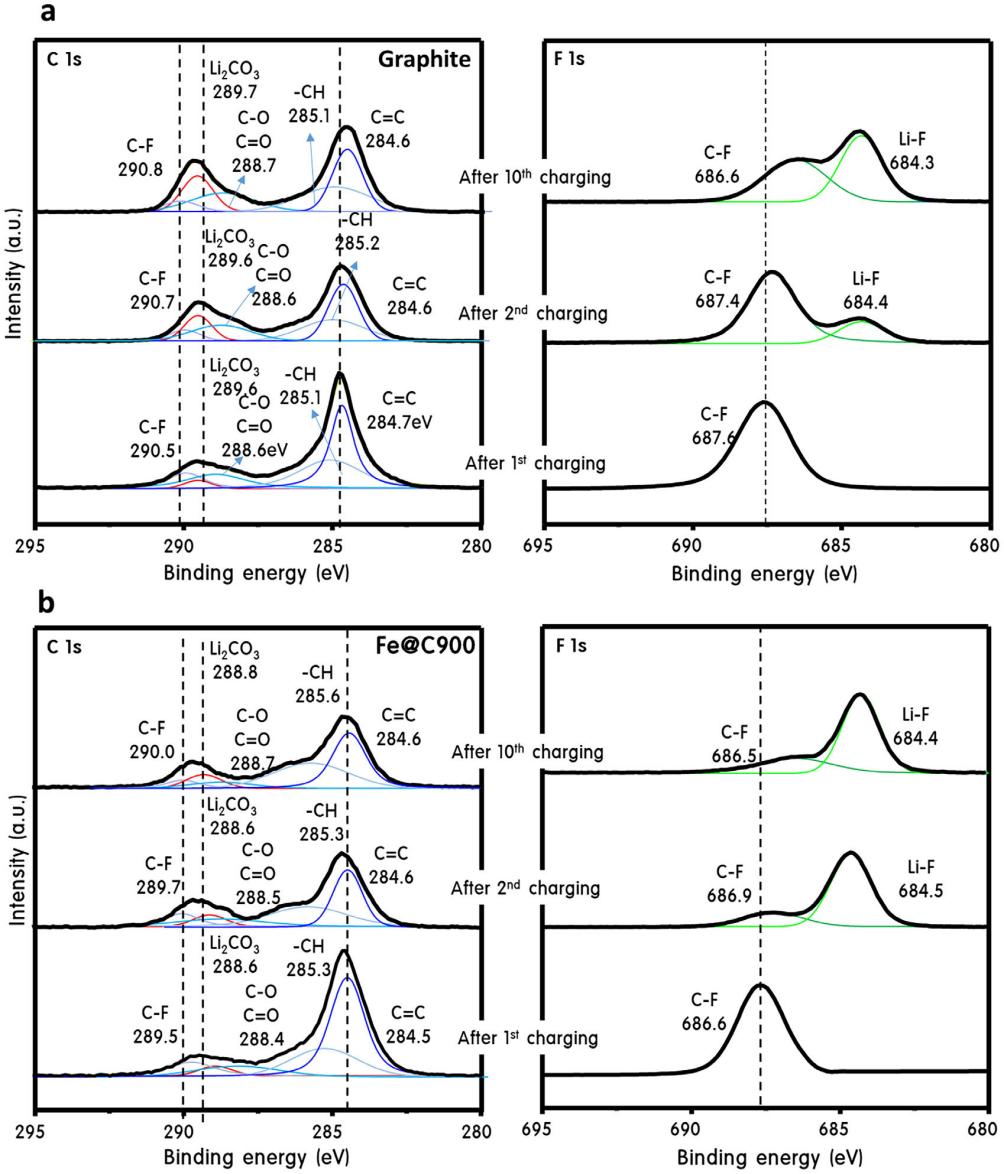
Unveiling SEI evolution: surface chemistry insights under cycling stress. a) C 1s and F 1s XPS spectra of Graphite anodes and b) C 1s and F 1s XPS spectra of Fe@C900 anodes.

The exceptional electrochemical stability of Fe@C900—achieved without conductive additives—is closely linked to the formation of a chemically uniform and structurally stable SEI. **Figure** **4** presents XPS analyses of both commercial graphite and Fe@C900 anodes after the 1^st^, 2^nd^, and 10^th^ cycles at 1C, offering critical insights into their respective SEI evolutions. In the C 1s spectra, both materials retained stable C═C (284.6–284.7 eV) and hydrocarbon peaks (285.0–285.2 eV), confirming carbon framework integrity during cycling. However, graphite showed a progressive increase in C═O, C─O, and Li₂CO₃ signals, indicating ongoing electrolyte decomposition and uncontrolled SEI thickening over time.^[^ ^26^ ^]^ In contrast, Fe@C900 maintained a constant intensity of Li₂CO₃ from the 1st to the 10th cycle, reflecting a self‐limiting and stable SEI formation in the initial cycle. Notably, Fe@C900 also preserved chemically stable oxygen‐containing groups (C═O, C─O) across all cycles, whereas graphite exhibited fluctuating intensities—further validating Fe@C900's superior SEI chemistry. The F 1s spectra further differentiate the two systems. In graphite, continuous decomposition of the PVdF binder led to increasing C─F signals (290.5–290.8 eV), resulting in excessive LiF accumulation.^[^ ^24^ ^]^ This overgrowth often disrupts interfacial uniformity and increases impedance. Conversely, Fe@C900 exhibited early stabilization of LiF content by the 2^nd^ cycle, with no further accumulation by the 10^th^ cycle, indicating a well‐regulated SEI growth window. These results confirm that Fe@C900 forms a thinner, more uniform, and compositionally stable SEI, minimizing parasitic reactions and suppressing electrolyte decomposition. The onion‐like graphitized carbon shell likely plays a dual role—physically buffering the interface and chemically stabilizing key SEI components (Li₂CO₃, LiF). Such a self‐regulating SEI mechanism underpins Fe@C900's long‐term cycling durability and fast‐charge capability, even in the absence of conductive additives. After 1000 cycles at 1C (Figure S2b, Supporting Information), the Fe@C900 electrode was retrieved, rinsed with solvent, and analyzed by HR‐TEM (Figure S5, Supporting Information). The onion‐like graphitic carbon shells remained structurally intact and uniform in thickness after long‐term cycling, with no signs of delamination, collapse, or cracking. The well‐preserved multilayered architecture highlights the outstanding electrochemical durability and structural resilience of the core–shell system, suggesting minimal interfacial degradation during extended operation.

### 2.4 Electrochemical Enhancement via Magnetic Field: Spin Alignment and Ionic Acceleration

**Figure 5:**
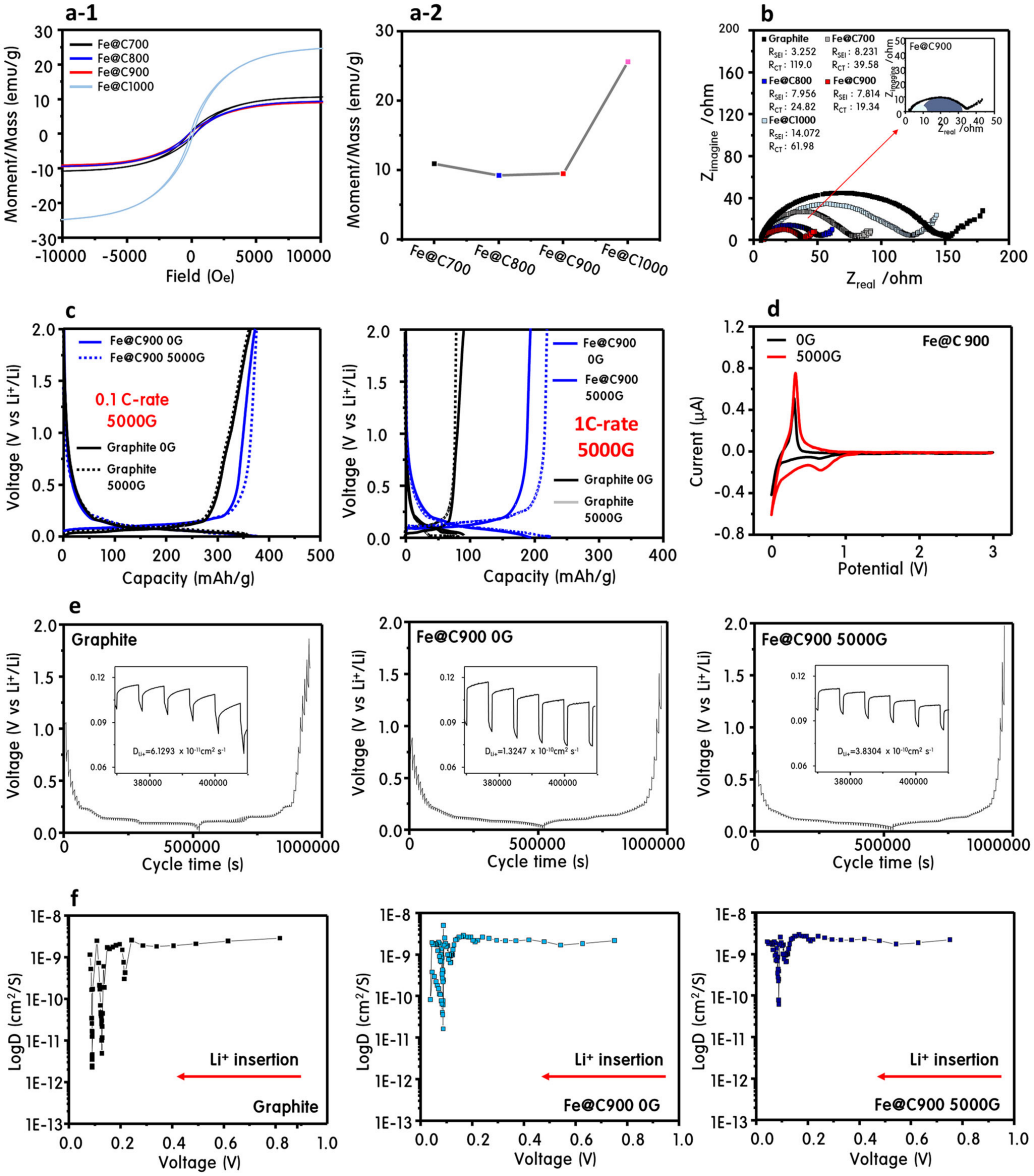
Magnetic and electrochemical performance of Fe@C_x_ anodes under external magnetic field conditions. (a‐1) Magnetic hysteresis loops of Fe@C_x_ samples obtained via VSM, (a‐2) Comparison of saturation magnetization among Fe@C_x_ samples, b) Nyquist plots obtained from EIS analysis for Graphite, Fe@C_x_ anodes, and Fe@C900 under 0G and 5000G magnetic fields, c,d) Charge–discharge profiles of Fe@C900 at 0.1 and 1 C under 0G and 5000G magnetic fields, e) CV curves of Fe@C900 at 0G and 5000G magnetic fields, and f) GITT voltage profiles and calculated lithium‐ion diffusion coefficients (*D*) for Graphite, Fe@C900 (0G), and Fe@C900 (5000G).

The unique ferromagnetic nature of Fe@C900 offers a previously untapped pathway to enhance lithium‐ion mobility via external magnetic fields. **Figure** **5** summarizes the magnetic, impedance, charge, and diffusion behaviors of Fe@Cx anodes, with Fe@C900 standing out as the magnetically responsive electrochemical system. Magnetization profiles obtained via VSM (Figure 5a‐1,a‐2) show that Fe@C700, Fe@C800, and Fe@C900 exhibit moderate saturation magnetizations (≈10 emu g⁻¹), while Fe@C1000 demonstrates a sharp increase (≈25 emu g⁻¹) due to partial Fe core exposure after carbon shell degradation. The encapsulated Fe cores in Fe@C900 suppress excessive magnetization, preserving spin uniformity without sacrificing ferromagnetic function. Electrochemical impedance spectroscopy (EIS, Figure 5b) reveals that Fe@C900 exhibits significantly lower charge‐transfer resistance (R_CT_) and improved Li⁺ mobility compared to graphite, even without additives. The diminished semicircle and steeper low‐frequency tail suggest enhanced ion kinetics and lower interfacial impedance.^[^ ^27^ ^]^ When tested under a 5000G magnetic field (Figure 5c), Fe@C900 exhibited a remarkable 150% increase in capacity at 1C, while graphite showed negligible change—indicating that the magnetic field actively promotes directional Li⁺ migration in Fe@C900. This improvement is attributed to spin alignment of the Fe cores (T₂g⁴Eg^2^), which likely induces localized magnetic ordering in the surrounding graphitic shells, smoothing Li⁺ pathways across the carbon network. Cyclic voltammetry profiles under 0G versus 5000G (Figure 5d) further confirm this enhancement. The magnetic field intensified redox peak currents and expanded the integrated area, indicating improved electrochemical reversibility. Importantly, no Fe oxidation peaks (≈1.9 V) were observed,^[^ ^28^ ^]^ suggesting that the magnetic field does not destabilize the Fe core or disrupt its encapsulation. Diffusion coefficients (D) obtained from GITT (Figure 5e,f) reinforce the magnetic enhancement. At ≈0.1 V, D increased from 6.13 × 10⁻¹¹ cm^2^ s^−1^ (graphite) to 1.32 × 10⁻¹⁰ cm^2^ s^−1^ (Fe@C900), and further to 3.83 × 10⁻¹⁰ cm^2^ s^−1^ under 5000G. This threefold boost is particularly pronounced in the 0.001–0.2 V range, where spin‐aligned Fe@C900 provides deep lithium intercalation through widened (002) interlayer spacing and magnetic field‐induced migration enhancement. Together, these results demonstrate that Fe@C900 operates as a magneto‐electrochemical system, wherein external magnetic fields synchronize spin states and accelerate Li⁺ ion kinetics—resulting in improved rate performance, deeper lithiation, and greater diffusion efficiency. This spin‐guided transport mechanism, enabled by the ferromagnetic core–shell design, positions Fe@C900 as a next‐generation anode for high‐power, intelligent battery systems.

### 2.5 In Situ XRD, Raman, and Field Mapping Reveal Lithium Insertion Dynamics

**Figure 6:**
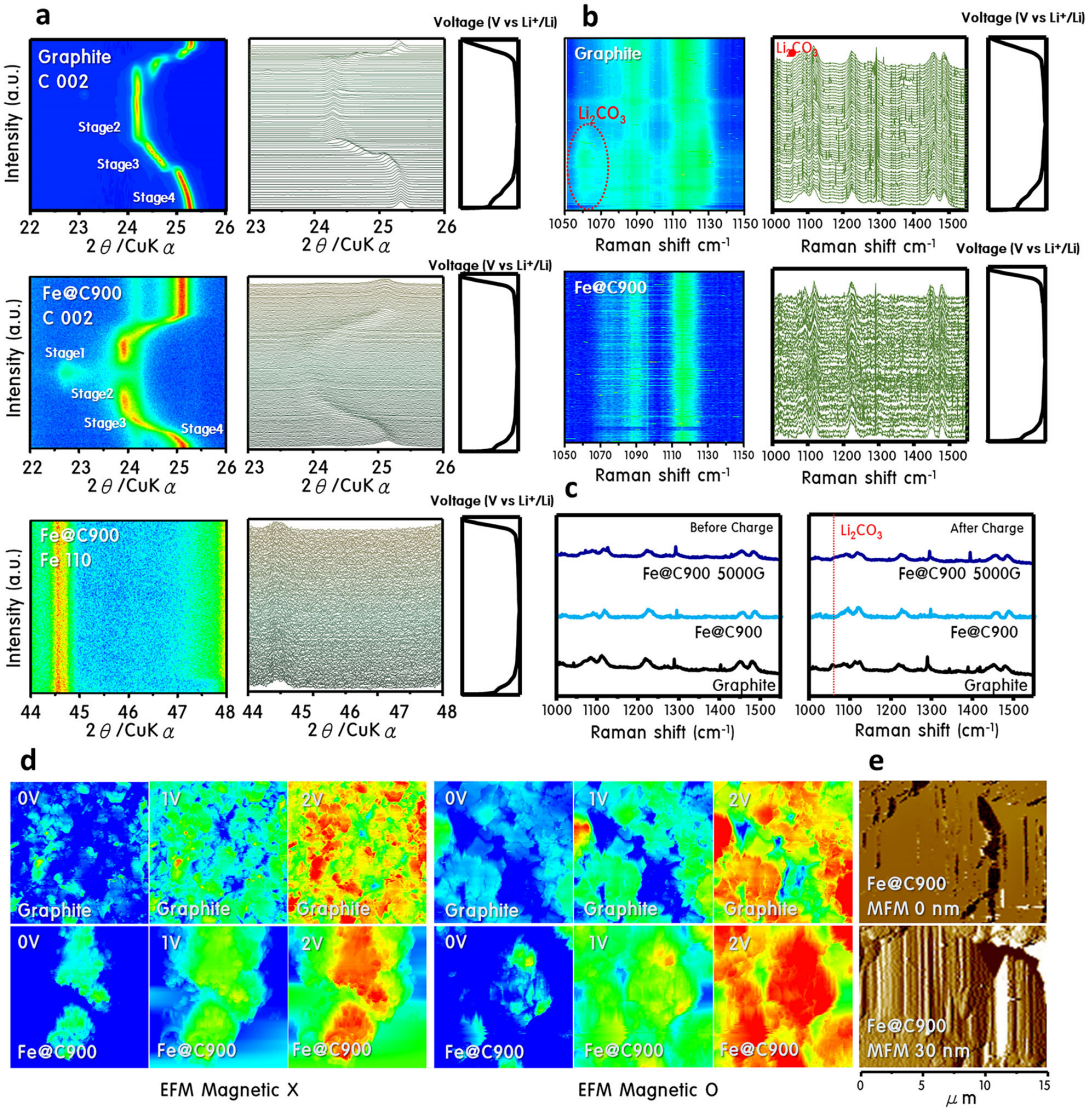
In situ structural and surface analysis of Graphite and Fe@C900 anodes during lithiation and delithiation processes. a) In situ XRD patterns showing structural changes in Graphite and Fe@C900 anodes during lithiation, b) In situ Raman analysis displaying Li₂CO₃ formation during lithiation, c) Raman spectra before and after lithiation, showing reduced chemical degradation and stable SEI formation on Fe@C900 compared to Graphite, d) Electrostatic Force Microscopy (EFM) images illustrating enhanced charge distribution and conductivity, and e) Magnetic Force Microscopy (MFM) image of Fe@C900 under a 5000G magnetic field.

To directly visualize lithium‐ion intercalation behavior and structural evolution during cycling, in situ XRD and Raman spectroscopy were employed, as shown in **Figure** **6**. These real‐time analyses uncover the distinct lithiation pathways and interfacial stability of Fe@C900 compared to commercial graphite. In situ XRD profiles (Figure 6a) collected during cycling at 0.2 C demonstrate that graphite undergoes a stepwise phase transition: Stage 4 (LiC₂₄) → Stage 3 (LiC₁₈) → Stage 2 (LiC₁₂), which stabilizes ≈300 mAh g⁻¹, as consistent with classical intercalation models.^[^ ^29^ ^]^ In contrast, Fe@C900 progresses further to Stage 1 (LiC₆), enabled by its expanded C (002) interlayer spacing, which facilitates deeper Li⁺ insertion and longer diffusion pathways. Notably, the Fe (110) peak remains unchanged throughout cycling, confirming that Fe does not participate in redox reactions and functions solely as a magnetic scaffold. In situ Raman measurements (Figure 6b) further validate interfacial stability. During charging, graphite shows a clear Li₂CO₃ Raman signal—an indicator of electrolyte decomposition and unstable SEI formation. Fe@C900, on the other hand, shows minimal Li₂CO₃ formation, indicating suppressed electrolyte degradation and early stabilization of the SEI. Post‐cycling Raman spectra (Figure 6c) confirm this, revealing heavy carbonate accumulation on graphite, whereas Fe@C900 maintains a clean surface, affirming its long‐term durability. To investigate electronic structure evolution under magnetic field influence, Electrostatic Force Microscopy (EFM) and Magnetic Force Microscopy (MFM) were conducted (Figure 6d,e). EFM reveals that Fe@C900 exhibits significantly higher local electron mobility than graphite (Figure 6d)—both under ambient and 5000G magnetic field conditions. The increased potential gradient under a magnetic field demonstrates magnetically enhanced electronic conductivity. MFM analysis (Figure 6e) visualizes well‐aligned magnetic domains (≈100 nm spacing) on Fe@C900 under a 5000G field, confirming spin ordering of Fe cores. These domains likely act as directional anchors for Li⁺, promoting smoother, spin‐aligned migration across the carbon shell network. Together, these in situ structural and surface mapping techniques confirm that Fe@C900's advanced carbon shell and internal ferromagnetic alignment collectively facilitate deep lithium intercalation, suppressed electrolyte degradation, and magnetic field‐assisted charge transport—a combination that underpins its exceptional electrochemical durability and rate capability.

### 2.6 Theory Meets Magnetism: Spin‐Aligned Diffusion Confirmed at the Atomic Scale

**Figure 7:**
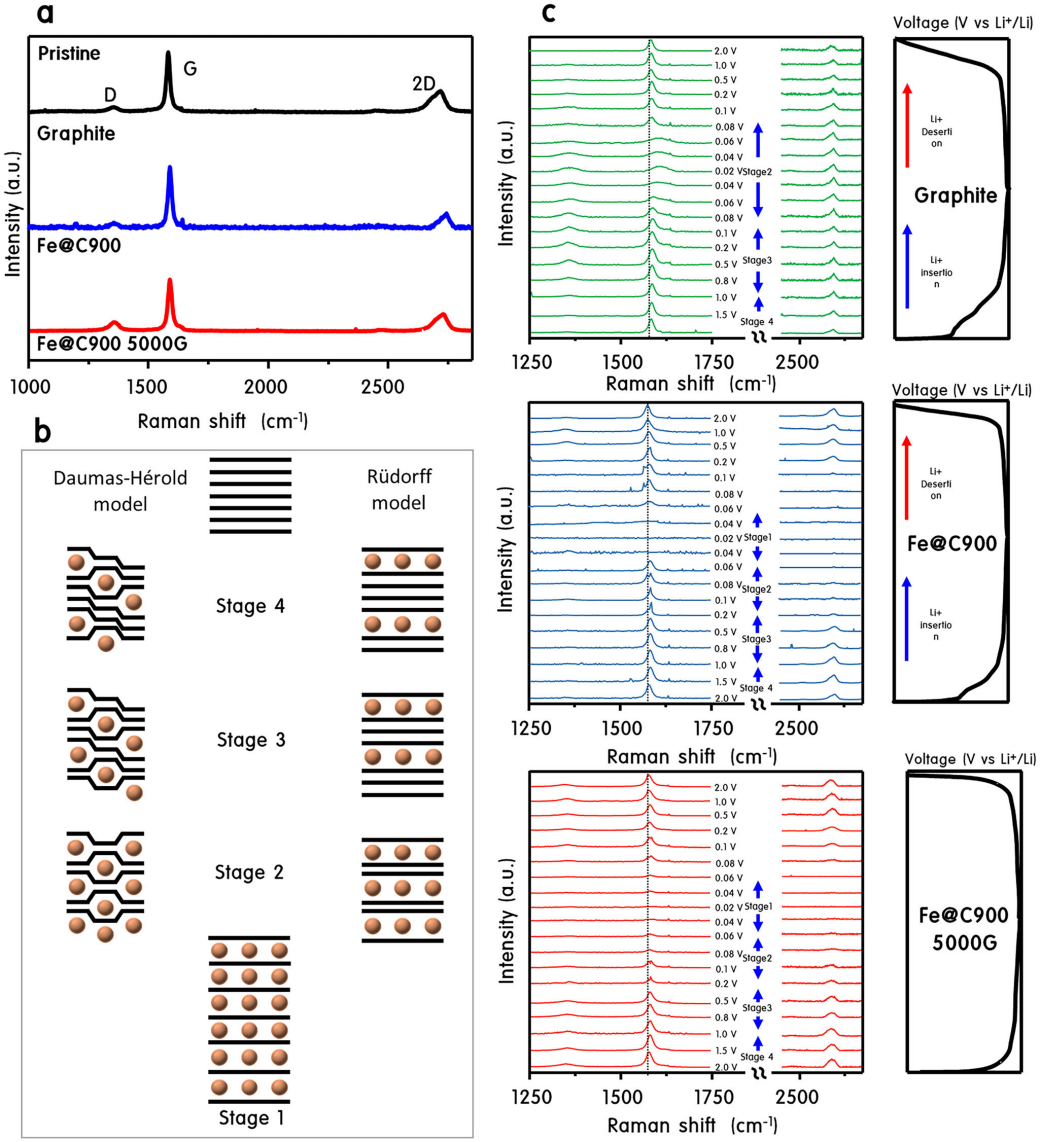
Raman analysis of Graphite, Fe@C900, and Fe@C900 under a 5000G magnetic field during lithiation and delithiation. a) Raman spectra of pristine Graphite, Fe@C900, and Fe@C900 (5000G) showing D‐band, G‐band, and 2D‐band peaks, b) Schematic representation of lithiation stages (Stage 4 to Stage 1) based on the Daumas‐Hérold and Rüdorff models. Fe@C900 and Fe@C900 (5000G) achieve complete lithiation to Stage 1, while Graphite progresses only to Stage 2, and c) In situ Raman spectra during lithiation showing the shift and broadening of the G‐band for Graphite, Fe@C900, and Fe@C900 (5000G).

To mechanistically validate the observed electrochemical enhancements under magnetic field and lithium intercalation conditions, DFT calculations and in situ Raman analysis were conducted. These atomic‐scale evaluations directly correlate spin alignment, carbon shell design, and lithium‐ion energetics in Fe@C900 versus graphite. DFT results (Figure S6, Supporting Information) highlight the thermodynamic advantage of Fe@C900 over graphite in lithium‐ion interactions. For graphite, the Gibbs free energy for Li⁺ adsorption on the surface is −12.87 eV, while insertion into the interlayer raises this to −11.42 eV, incurring a 1.45 eV penalty due to lattice strain and distortion.^[^ ^30^ ^]^ In Fe@C900, however, the Li⁺ adsorption energy is significantly more favorable at −24.14 eV, and insertion only marginally increases to −22.85 eV (ΔE = 1.29 eV). This thermodynamic profile suggests that Fe@C900 offers both stronger lithium affinity and reduced energy barriers for insertion—consistent with its wider (002) interlayer spacing observed via XRD. The enhanced Li⁺ adsorption is attributed to Fe@C900's unique architecture, where conductive metallic Fe cores enhance electronic density, while encapsulating graphitic layers stabilize the interface. This synergy reinforces both charge density and electrochemical resilience during cycling. In situ Raman spectroscopy (**Figure** **7**) further visualizes these atomic‐scale phenomena. Spectra collected under a horizontal cell configuration reveal that all samples exhibit D (≈1330 cm⁻¹), G (≈1580 cm⁻¹), and 2D (≈2670 cm⁻¹) bands at open‐circuit voltage (OCP) (Figure 7a). Fe@C900 and its 5000G variant display sharper, more defined peaks than graphite, indicating superior structural uniformity. During discharge, graphite shows lithiation up to Stage 2, while Fe@C900 and Fe@C900@5000G reach Stage 1, signifying full intercalation (Figure 7b)—corroborating in situ XRD and DFT data. Figure 7c shows that the G‐band undergoes a blue shift and broadening during lithiation, corresponding to reduced electron density and altered sp^2^ bonding. In graphite, the G‐band intensity decays, while the D‐band grows, resulting in a rising ID/IG ratio—signaling bond disorder and interlayer instability. By contrast, Fe@C900 exhibits a sharp decrease in D‐band intensity near 0.1 V, especially under magnetic field conditions, indicative of efficient and stable SEI formation. This contrasts with graphite, where Li₂CO₃ formation and structural distortions persist. Moreover, the 2D band undergoes a similar blue shift, reflecting in‐plane lattice deformation during deep lithiation. For graphite, this shift coincides with rapid performance decay and interfacial degradation, as predicted by the Daumas–Herold model.^[^ ^31^ ^]^ Fe@C900 resists this degradation due to its flexible carbon layers and ferromagnetic core, which aligns spin orientation and lowers Li⁺ insertion barriers under magnetic field. Collectively, the theoretical and spectroscopic analyses converge to confirm that Fe@C900 offers stronger Li⁺ binding, lower insertion energy, wider interlayer channels, and magnetic field‐responsive spin alignment. These atomic‐scale features culminate in its exceptional electrochemical performance and stability—even in the absence of conductive additives or artificial coatings.

## 3 Conclusion

This study introduces a sustainable and scalable upcycling strategy that transforms waste‐derived Fe‐based methane pyrolysis catalysts into high‐performance, conductor‐free anode materials for lithium‐ion batteries (LIBs). Among the engineered variants, Fe@C900—fabricated at 900 °C—demonstrates exceptional structural and electrochemical properties, surpassing commercial graphite in capacity, cycling stability, and rate performance. The superior behavior of Fe@C900 stems from its highly graphitized, onion‐like carbon shells with expanded (002) interlayer spacing, which facilitates rapid and deep Li⁺ intercalation. DFT calculations support the experimental findings, revealing favorable adsorption energies and reduced insertion barriers compared to graphite. These attributes collectively enable robust lithium storage and long‐term cycling resilience. Critically, Fe@C900 stabilizes the SEI, forming a thinner, chemically uniform layer that resists degradation. Unlike graphite, which suffers from continuous SEI thickening and Li₂CO₃ accumulation, Fe@C900 maintains a stable interfacial chemistry during extended cycling, as confirmed by XPS and in situ Raman analyses. Notably, the application of an external magnetic field (5000G) significantly enhances lithium‐ion transport via spin alignment within the ferromagnetic Fe core. GITT, EIS, EFM, and MFM data reveal that magnetic modulation improves charge transfer kinetics and ionic diffusion, elevating capacity by up to 150% compared to graphite under identical conditions. Together, these findings define Fe@C900 as a new class of magneto‐functional anodes that leverage ferromagnetism and graphitic nanostructures to enable high‐rate, additive‐free LIB performance. This work pioneers a circular‐material design paradigm and introduces spin‐guided lithium transport as a promising frontier in electrochemical energy storage.

## Conflict of Interest

The authors declare no conflict of interest.

## Supporting information



Supporting Information

## Data Availability

The data that support the findings of this study are available from the corresponding author upon reasonable request.
