# Supplementary material for: Spin‐Orchestrated Lithium Diffusion in Reforged Ferromagnetic Fe@C Anodes
Source: Adv Sci (Weinh). 2025 Jul 2;12(36):e06133. doi: 10.1002/advs.202506133 (PMC12463094; doi:10.1002/advs.202506133)
Supplement: Supplementary file 1 — Supporting Information [file ADVS-12-e06133-s001.docx]

Supplementary Information

**Spin-Orchestrated Lithium Diffusion in Reforged Ferromagnetic Fe@C Anodes**

**Myeong Seok Goh^1^, Hyunsub Shin^1^, Jaehun Lee^1^, No-Kuk Park^2^, Joonwoo Kim^3^, Sang Woo Joo^4^, Ki Hyeon Kim^5,*^, Misook Kang^1,**^**

*^1^Department of Chemistry, College of Natural Sciences, Yeungnam University, Gyeongsan, Gyeongbuk 38541, Republic of Korea*

*^2^Institute of Clean Technology, Yeungnam University, Gyeongsan, Gyeongbuk 38541, Republic of Korea*

*^3^Industrial Gas Research Group, Research Institute of Industrial Science & Technology (RIST), 67 Cheongam-ro, Nam-gu, Pohang, Gyeongbuk 37673, Republic of Korea*

*^4^School of Mechanical Engineering, Yeungnam University, Gyeongsan, Gyeongbuk 38541, Republic of Korea*

*^5^Department of Physics, College of Natural Sciences, Yeungnam University, Gyeongsan 38541, Republic of Korea*

^1^They contributed equally to this study.

^*,**^Corresponding authors: [kee1@ynu.ac.kr](mailto:kee1@ynu.ac.kr), mskang@ynu.ac.kr

**Material and methods**

**Chemicals**

- Iron precursor: Iron(III) nitrate nonahydrate (Fe(NO₃)₃·9H₂O, 98%, Junsei Chemical Co., Tokyo, Japan)
- Electrolyte: 1.0 M LiPF₆ in EC:DEC = 1:1 (v/v), PanaX etxc. Co., Korea
- Lithium metal: Foil type (≥99.9%), Honjo Chemical, Japan
- Binder: Polyvinylidene Fluoride (PVDF, 99.5%, MTI Corp., USA)
- Conducting material: Super P (99+%, Thermo Scientific, USA) (only used in graphite reference electrode, not in Fe@C900)
- Solvent for slurry: N-Methyl-2-pyrrolidone (NMP, ≥99%, Sigma-Aldrich, USA)
- Post-cycling washing solvent: Dimethyl carbonate (DMC, anhydrous, ≥99%, Sigma-Aldrich, USA)
- Gases:
   - Methane (CH₄, 99.999%, Pangang Industrial Gas, Korea)
   - Argon (Ar, 99.999%)
   - Hydrogen (H₂, 99.999%)

**Iron oxide catalyst used for methane pyrolysis and the Fe@C_x_ waste catalyst**

To produce turquoise hydrogen, methane pyrolysis was conducted using Fe oxide catalysts. Iron(III) nitrate nonahydrate (Fe(NO₃)₃·9H₂O, 98%, Junsei Co., Tokyo, Japan) was used as the precursor for the catalyst. The precursor was dissolved in a 1:1 (v/v) mixture of distilled water and ethanol in a 1 L beaker and stirred continuously for 12 hours to ensure complete dissolution and homogeneity. The solution was gradually heated to 80°C to evaporate the solvent, resulting in a uniform powder. The obtained powder was dried in an oven at 120°C for 12 hours to eliminate residual moisture. The dried powder was then sintered at 1000°C for 6 hours in an air atmosphere to produce crystalline Fe₂O₃. The resulting crystalline material was finely ground using an agate mortar and sieved through a 100 µm mesh. Only the fine particles that passed through the mesh were collected and used as catalysts for methane pyrolysis.

**Methane pyrolysis and recovery of Fe@C_x_ catalysts**

Methane pyrolysis was conducted by filling 1.5 g of the Fe₂O₃ powder prepared as described above into a quartz reactor with an inner diameter of 2.0 cm, an outer diameter of 2.2 cm, and a length of 60.0 cm, as shown in Scheme S1. Methane pyrolysis is a process that produces hydrogen by thermally cracking methane on a catalyst without the addition of oxygen or water, depositing solid carbon on the catalyst surface instead of forming carbon oxides (CO, CO₂).^[1]^ The hydrogen produced during this process is classified as turquoise hydrogen, a renewable energy source that is economical and eliminates the need for carbon capture and storage. As a result, many countries are actively exploring this method for hydrogen production.

This endothermic process occurs at high temperatures exceeding 700°C, as represented by the following reaction equation:^[2]^ CH₄(g) → C(s) + 2H₂(g), ΔH⁰net = 74.8 kJ/molCH₄. Transition metal-based catalysts are commonly used to lower the activation energy of this reaction. Ni-based catalysts, for instance, effectively reduce the reaction temperature but suffer from rapid deactivation due to strong carbon deposition on active sites during methane decomposition, along with high costs.^[3]^ Consequently, Fe-based catalysts, which are more cost-effective, are widely used in industrial applications and were also employed in this study. The Fe₂O₃ catalyst was pretreated with a 5% H₂/Ar gas mixture at 900°C for 2 hours for reduction before use. After stabilization at the appropriate reaction temperature for 1 hour, methane pyrolysis was performed. Pure methane gas (99.999%) was introduced into the system, with an Ar carrier gas maintaining a methane flow rate of 50 mL/min. The weight hourly space velocity (WHSV) was fixed at 2000 mL/h·g. Methane pyrolysis reactions were conducted at varying temperatures of 700°C, 800°C, 900°C, and 1000°C, with a constant reaction time of 15 hours. During the reaction, methane conversion rates and hydrogen selectivity were monitored hourly. After the 15-hour reaction, the spent Fe@C_x_ catalysts (where x denotes the reaction temperature) were recovered for use as active materials in LIB anodes. The recovered Fe@C_x_ catalysts were named Fe@C700, Fe@C800, Fe@C900, and Fe@C1000, corresponding to the methane pyrolysis temperatures of 700°C, 800°C, 900°C, and 1000°C, respectively.

**Characterization of Fe@C_x_ anode materials**

The crystalline structures of Fe@C700, Fe@C800, Fe@C900, and Fe@C1000 anode materials were characterized using X-ray diffraction (XRD, Miniflex, Rigaku, Tokyo, Japan) over a 2θ range of 10–90°. Quantitative and qualitative analyses of carbon content were performed using thermogravimetric analysis (TGA, N-1000, SINCO, Korea) and energy-dispersive spectroscopy (EDS). For TGA, 15.0 mg of each sample was heated from 100°C to 1000°C at a rate of 10°C/min under a 100 mL/min air flow to determine the precise carbon content in the Fe@C_x_ anode materials. The crystallinity of the deposited carbon was analyzed using Raman spectroscopy (Horiba Xplora Plus) within the spectral range of 500–2000 cm⁻¹.

The surface morphologies of Fe@C_x_ anode materials synthesized at various methane pyrolysis temperatures were examined using scanning electron microscopy (SEM, S-4800, HITACHI, Japan), enabling observation of carbon growth patterns. The specific surface areas of the Fe@C_x_ materials were measured via nitrogen adsorption-desorption isotherms using the Brunauer–Emmett–Teller (BET) method on a Micromeritics ASAP 2000 system. High-resolution transmission electron microscopy (HR-TEM, Titan G2 STEM, FEI Company, USA) was employed to capture lattice images and selected area electron diffraction (SAED) patterns for the anode material that exhibited the most favorable electrochemical performance.

To elucidate the carbon growth mechanism, a focused ion beam system (FIB-TEM, Thermo Fisher Scientific, Helios 5 CX) was used. Additionally, X-ray photoelectron spectroscopy (XPS, AXIS-NOVA, Kratos Inc., USA) analyzed the elemental composition, oxidation states, and SEI (solid electrolyte interphase) layer formation in Fe@C_x_ anode materials before and after reactions. For the best-performing Fe@C_x_ anode and a commercial graphite anode, XPS was conducted after the 1^st^, 2n^d^, and 10^th^ charge/discharge cycles to infer SEI layer development. After cycling, cells were disassembled, and the electrodes were washed with dimethyl carbonate (DMC) to remove residual Li salts. The cleaned electrodes were vacuum-dried at 25°C and subsequently prepared for XPS analysis.

**Cell fabrication and electrochemical property evaluation**

To fabricate conductive additive-free LIB half-cells using Fe@C_x_ anode materials, a slurry was prepared by mixing N-methyl-2-pyrrolidone (NMP) as the solvent, 90.0 wt.% Fe@C_x_ as the active material, and 10.0 wt.% polyvinylidene fluoride (PVDF) as the binder in an agate mortar. The mixture was thoroughly ground to achieve a homogeneous consistency. No conductive additives were used in the preparation. The prepared slurry was evenly applied onto a copper foil using a microfilm applicator (doctor blade) to form a 50 µm-thick coating. The average mass loading of the active materials was approximately 1.06 mg cm⁻². The coated electrodes were vacuum-dried at 120°C to remove the NMP solvent and subsequently pressed using a roll press. Circular electrodes with a diameter of 11.0 mm were punched out and vacuum-dried again at 110°C for 6 hours to eliminate any remaining moisture and NMP. The dried electrodes were transferred into an Ar (99.999%) atmosphere glove box for cell assembly. Half-cells were assembled by stacking components in the following order: can, Fe@C_x_ anode, electrolyte (1.0 M LiPF₆ in EC:DEC = 1:1, PanaX etxc. Co., Busan, Korea, 80 μL per cell), polyethylene separator (pre-soaked in the electrolyte for 24 hours), gasket, lithium metal chip (cathode), spacer, spring, and cap. The assembled half-cells were sealed for subsequent electrochemical evaluation.

Electrochemical measurements were carried out at room temperature (25°C) and atmospheric pressure. Charge-discharge capacities, cycle life, recovery rate, and cyclic voltammetry (CV) tests were performed in the voltage range of 0.001–2.0 V (vs. Li/Li⁺) using battery testing equipment (WBCS3000, WonATech Co., Seoul, Korea). Charge-discharge tests were conducted at a 0.1 C-rate, while recovery rate tests were performed sequentially at 0.1, 0.5, 1, 2, 5, and 10 C-rates, with each C-rate repeated five times before returning to 0.1 C-rate for final evaluation.

The internal resistance of the coin cells was measured using an electrochemical impedance spectroscopy system (IVIUMnSTAT, Ivium Technologies, Netherlands). Measurements were performed with an AC signal amplitude of 50.0 mV across a frequency range from 100 Hz to 0.01 Hz.

**Evaluation of electrochemical performance of Fe@C_x_ anode materials under an external magnetic field**

The electrochemical performance of Fe@C_x_ anode materials was evaluated under an external magnetic field by vertically aligning the electrodes parallel to the magnetic field, as shown in Scheme S1. The magnetic field strength was set at 5000 G, and a magnetic field generator (KR TECH, Magnetic & Vacuum, Tailor-Made) with an output of 30 V and 30 A was used. Recognizing that the relative orientation between the magnetic field and the battery could significantly impact electrochemical behavior, the positioning was carefully fixed throughout the experiments. In horizontally mounted electrodes (A), the magnetic field and electric field intersected perpendicularly, generating Lorentz forces that distorted the migration paths of lithium ions. This distortion increased the ionic resistance within the electrodes, elongating the migration pathways and subsequently degrading electrochemical performance. In contrast, vertically mounted electrodes aligned the magnetic and electric fields in parallel (B), eliminating the influence of Lorentz forces. This configuration allowed for optimized lithium ion migration paths, enabling a focused investigation of the magnetic field’s effects on the material properties and structure of the Fe@C_x_ anodes. Moreover, the vertical alignment ensured that the spin directions of the Fe cores aligned complementarily with the external magnetic field, irrespective of the lithium ion migration direction. This arrangement isolated the intrinsic effects of the magnetic field on the electrochemical properties of the material. Preliminary tests revealed that horizontally mounted electrodes experienced performance degradation due to the cancellation effects between the Fe core spins and the external magnetic field.

To address these challenges, this study employed a vertical electrode alignment to ensure the magnetic field influenced only the spin orientation of the Fe cores, without affecting the lithium ion migration path. This setup enabled a precise and unambiguous analysis of the magnetic field’s role in enhancing the structural and electrochemical characteristics of Fe@C_x_ anode materials.

**DFT calculation methodology**

First-principles calculations based on density functional theory (DFT) were performed using the Vienna Ab-initio Simulation Package (VASP). The exchange-correlation functional was treated using the generalized gradient approximation (GGA) with the Perdew-Burke-Ernzerhof (PBE) formulation. The projector augmented wave (PAW) method was employed to describe the core–valence electron interaction. A plane-wave cutoff energy of 500 eV was used, and convergence criteria were set to 10⁻⁴ eV for energy and 0.02 eV/Å for force. All structures were fully relaxed before calculating adsorption and insertion energies for Li⁺ ions on both Graphite and Fe@C900 surfaces. The Gibbs free energies were derived from total energy differences, with vibrational contributions neglected due to their minimal effect at room temperature.

[1] Alhamed, H., Behar, O., Saxena, S., Angikath, F., Nagaraja, S., Yousry, A., Das, R., Altmann, T., Dally, B., Sarathy, S. M., From methane to hydrogen: A comprehensive review to assess the efficiency and potential of turquoise hydrogen technologies, *Int. J. Hydrogen Energy* 2024, 68, 635–662. https://doi.org/10.1016/j.ijhydene.2023.10.233.

[2] Bayat, N., Rezaei, M., Meshkani, F., Methane decomposition over Ni–Fe/Al₂O₃ catalysts for production of COₓ-free hydrogen and carbon nanofiber, *Int. J. Hydrogen Energy* 2016, 41, 1574–1584. https://doi.org/10.1016/j.ijhydene.2015.11.023.

[3] Li, J., Croiset, E., Ricardez-Sandoval, L., Effect of carbon on the Ni catalyzed methane cracking reaction: A DFT study, *Appl. Surf. Sci.* 2014, 311, 435–442. https://doi.org/10.1016/j.apsusc.2014.05.100.


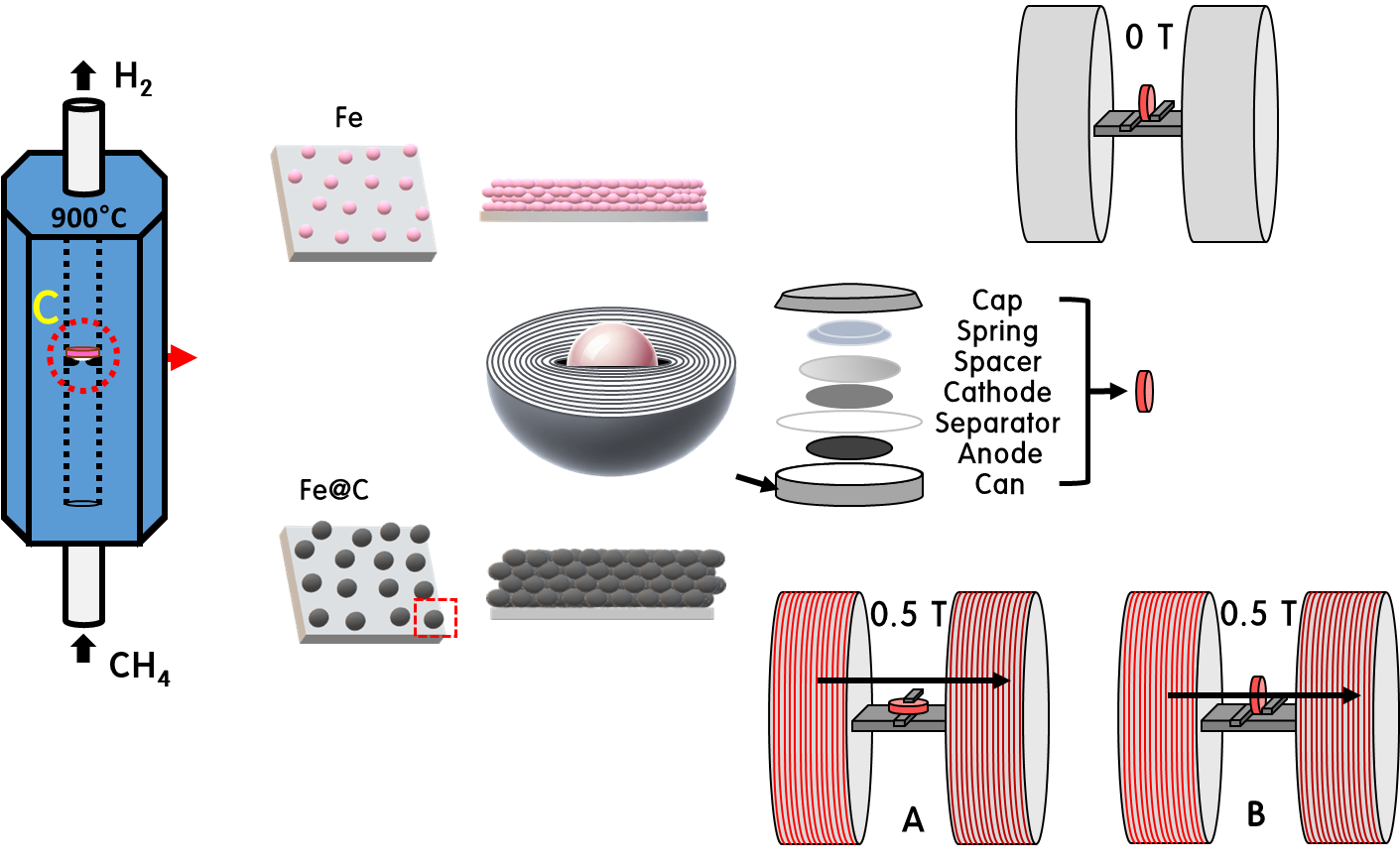


**Scheme S1. Schematic representation of the synthesis, assembly, and testing process of Fe@C materials.**

The process begins with methane pyrolysis at 900°C, where Fe catalysts are reduced and onion-like carbon shells are deposited to form Fe@C materials. The typical half-cell configuration includes an anode, separator, cathode, and other cell components. Magnetic field conditions (0 T and 0.5 T) were applied only during electrode testing to evaluate the influence of the magnetic field on lithium-ion diffusion and charge transfer performance.


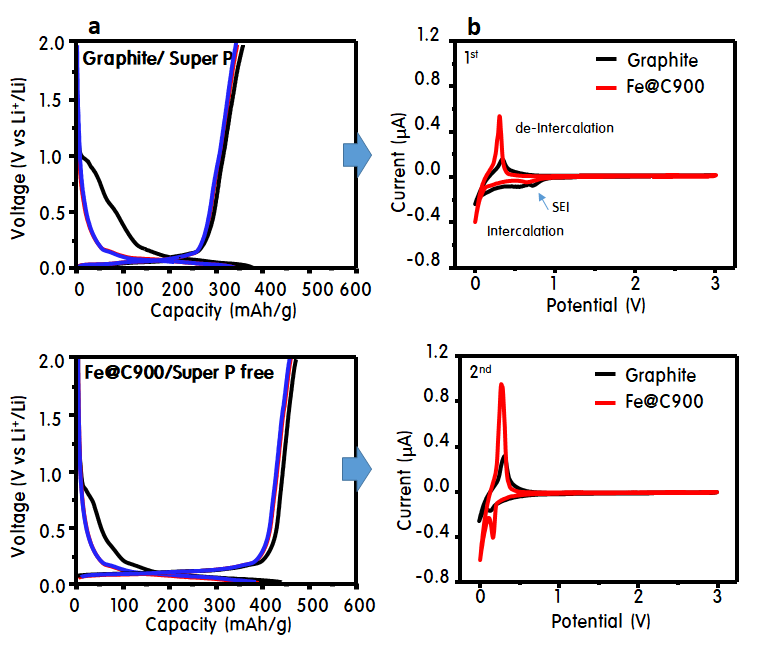


**Figure S1. Electrochemical performance comparison of Graphite with Super P and Fe@C900 without conductive additives.** (a) Voltage vs. capacity profiles of Graphite with Super P (top) and Fe@C900 without conductive additives (bottom) during initial charge-discharge cycles and (b) Cyclic voltammetry (CV) profiles of Graphite and Fe@C900 for the first and second cycles.


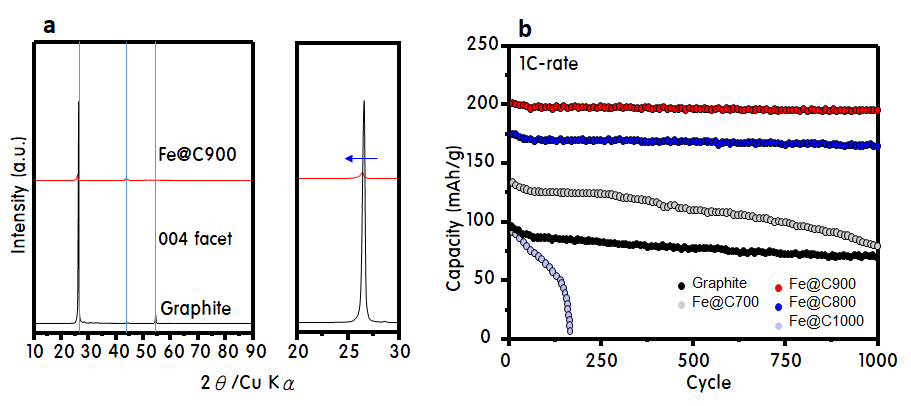


**Figure S2. Structural and cycling stability comparison between Graphite and Fe@Cₓ samples.** (a) XRD patterns of Graphite and Fe@C900, highlighting the broader interlayer spacing (002 facet) in Fe@C900 compared to Graphite and (b) Long-term cycling performance of Graphite and Fe@Cₓ samples at a 1C-rate.


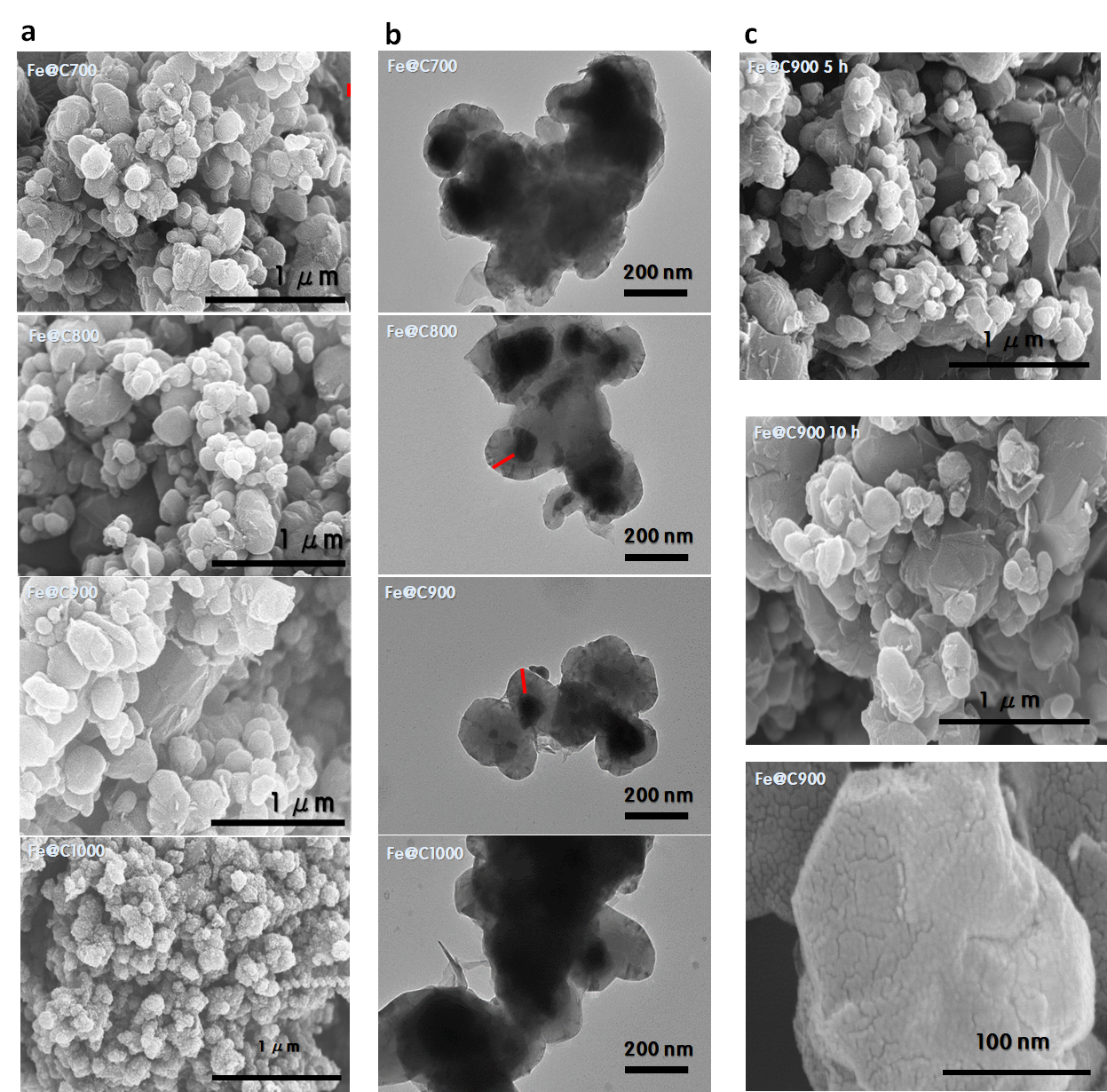


**Figure S3. Morphological analysis of Fe@Cₓ catalysts via SEM and TEM.** (a) SEM images of Fe@C700, Fe@C800, Fe@C900, and Fe@C1000 samples after methane pyrolysis, (b) TEM images of Fe@Cₓ samples highlighting onion-like carbon shells encapsulating Fe cores, and (c) SEM images of Fe@C900 obtained after 5, 10, and 15 hours of methane pyrolysis.


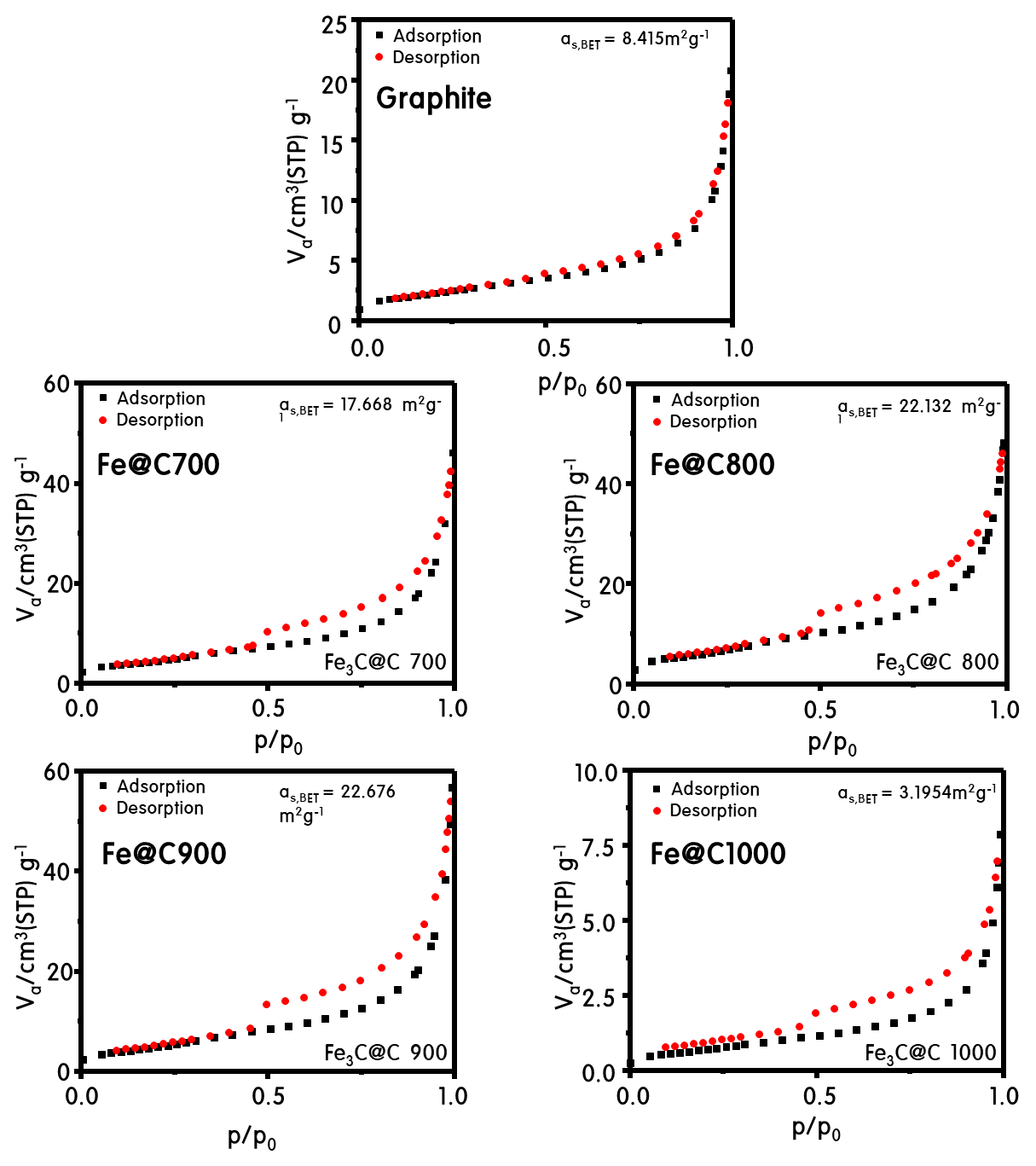


**Figure S4. Nitrogen adsorption-desorption isotherms of Graphite and Fe@Cₓ samples for BET surface area analysis.** The BET surface areas for Fe@C700, Fe@C800, Fe@C900, and Fe@C1000 are 17.668 m² g⁻¹, 22.132 m² g⁻¹, 22.676 m² g⁻¹, and 3.195 m² g⁻¹, respectively.


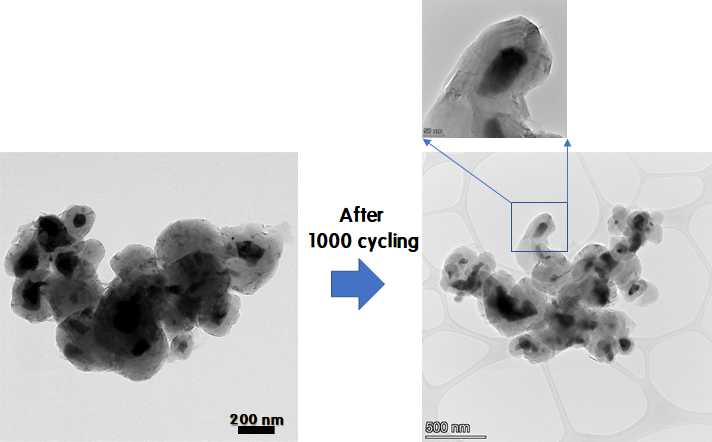


**Figure S5. Post-cycling HR-TEM analysis of Fe@C900 after 1000 charge/discharge cycles at 1C.**


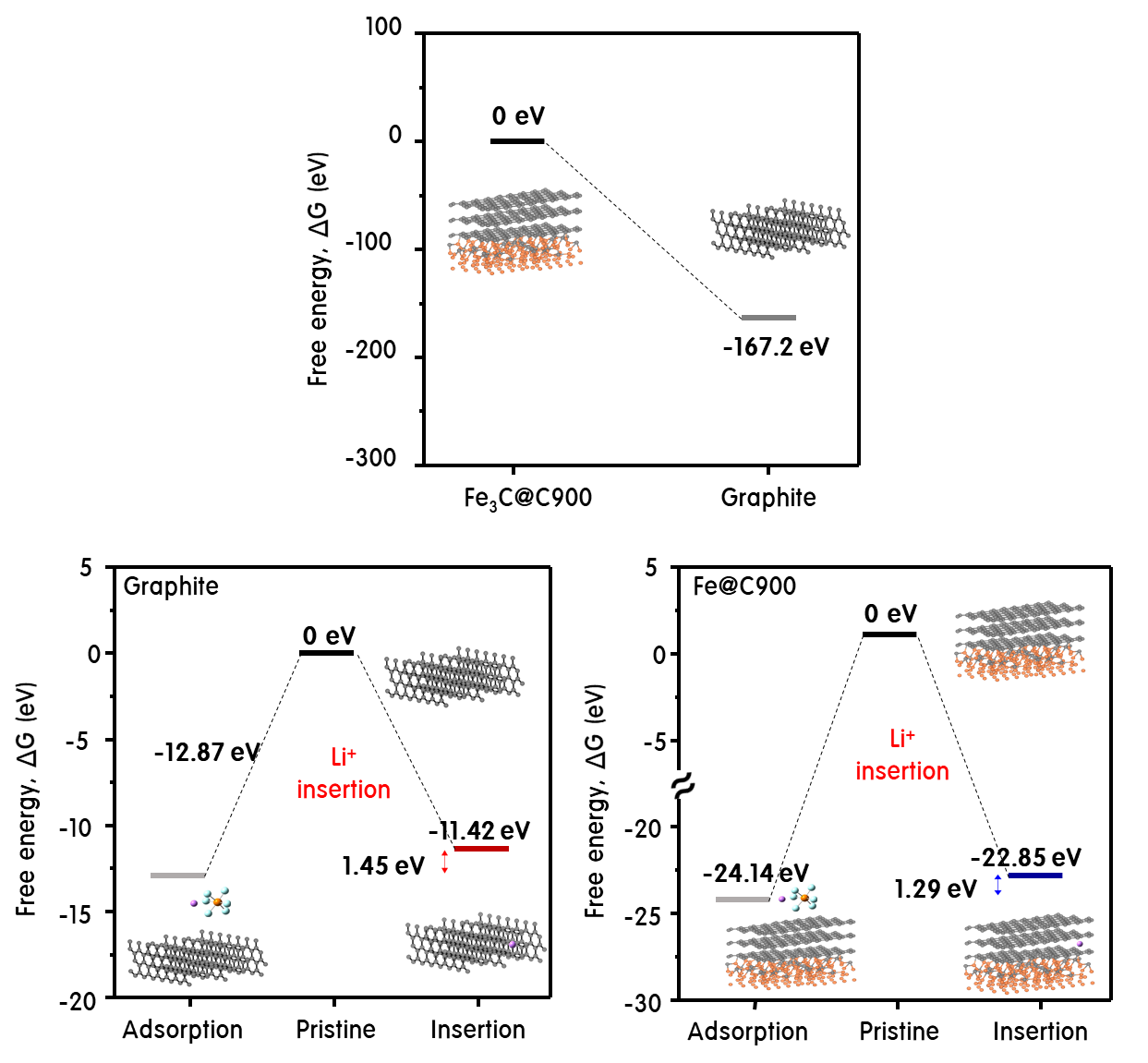


**Figure S6. Density functional theory (DFT) calculations comparing the free energy changes (ΔG) for lithium-ion adsorption and insertion in Graphite and Fe@C900.** For Fe@C900, lithium adsorption exhibits a much lower free energy of −24.14 eV, with a minor increase to −22.85 eV during insertion, resulting in a reduced energy barrier of +1.29 eV. These results demonstrate Fe@C900's superior lithium-ion interaction and structural stability compared to Graphite.
